# Characterization of the *Shewanella oneidensis* Fur gene: roles in iron and acid tolerance response

**DOI:** 10.1186/1471-2164-9-S1-S11

**Published:** 2008-03-20

**Authors:** Yunfeng Yang, Daniel P Harris, Feng Luo, Liyou Wu, Andrea B Parsons, Anthony V Palumbo, Jizhong Zhou

**Affiliations:** 1 Biosciences Division, Oak Ridge National Laboratory, Oak Ridge, TN 37831, USA; 2 School of Computing, Clemson University, Clemson, SC 29634, USA; 3 Institute for Environmental Genomics, and Department of Botany and Microbiology, University of Oklahoma, Norman, OK 73019, USA

## Abstract

**Background:**

Iron homeostasis is a key metabolism for most organisms. In many bacterial species, coordinate regulation of iron homeostasis depends on the protein product of a Fur gene. Fur also plays roles in virulence, acid tolerance, redox-stress responses, flagella chemotaxis and metabolic pathways.

**Results:**

We conducted physiological and transcriptomic studies to characterize Fur in *Shewanella oneidensis*, with regard to its roles in iron and acid tolerance response. A *S. oneidensisfur* deletion mutant was defective in growth under iron-abundant or acidic environment. However, it coped with iron depletion better than the wild-type strain MR-1. Further gene expression studies by microarray of the *fur* mutant confirmed previous findings that iron uptake genes were highly de-repressed in the mutant. Intriguingly, a large number of genes involved in energy metabolism were iron-responsive but Fur-independent, suggesting an intimate relationship of energy metabolism to iron response, but not to Fur. Further characterization of these genes in energy metabolism suggested that they might be controlled by transcriptional factor Crp, as shown by an enriched motif searching algorithm in the corresponding cluster of a gene co-expression network.

**Conclusion:**

This work demonstrates that *S. oneidensis* Fur is involved in iron acquisition and acid tolerance response. In addition, analyzing genome-wide transcriptional profiles provides useful information for the characterization of Fur and iron response in *S. oneidensis*.

## Background

Iron is the second most abundant element in the earth's crust and an essential micronutrient for virtually all organisms. The redox features of iron make it a useful cofactor for many proteins functioning in respiration, photosynthesis, nitrogen fixation and DNA biosynthesis, to name a few. However, iron also forms highly reactive oxygen species that can impose severe cellular damage [1,2]. Hence, intracellular iron concentration must be tightly controlled. In *E. coli*, iron-binding transporters located in the outer and inner membranes are induced to actively import iron under iron-limited conditions [3]. To facilitate the capture of iron, *E. coli* produces and releases the iron-chelating siderophores to the surrounding environment. Also, the iron storage proteins (ferritin and bacterioferritin) and non-essential iron-using proteins are repressed to release their sequestered iron [4]. When iron is sufficient, the iron uptake and homeostatic systems are coordinately regulated by a global transcriptional factor Fur (the ferric uptake regulator), which complexes with Fe^2+^ and represses the transcription of a diverse array of genes [5]. The DNA target recognized by Fe^2+^-Fur is a 19-bp inverted repeat sequence of GATAATGATAATCATTATC [6]. The binding of Fur to this motif in the promoter regions of target genes effectively prevents the recruitment of the RNA polymerase holoenzyme and thus represses transcription [7,8]. Although the major role of Fur is to regulate genes involved in iron homeostasis systems, Fur has also been demonstrated to be a pleiotropic transcriptional factor. As such, it functions in a variety of cellular processes including redox-stress responses, flagella chemotaxis, metabolic pathways, acid tolerance and virulence [9-11]. Indeed, it has been estimated that Fur directly controls the expression of over ninety genes in *E. coli*[11].

Maintaining iron homeostasis is of particular interest for a γ-proteobacterium *Shewanella oneidensis* for several reasons. First, *S. oneidensis* is capable of respiring with a variety of electron acceptors including oxygen, glycine, nitrate, nitrite, thiosulfate, elemental sulfur, fumarate, dimethyl sulfoxide (DMSO), trimethylamine N-oxide (TMAO), Fe(III), Mn(IV), Co(III), U(VI), Cr(VI) and Tc(VII) [12-16]. Unlike most other γ-proteobacteria, *S. oneidensis* is capable of respiring Fe(III). Therefore, iron serves as not only a protein cofactor but also an important electron acceptor for the bacterium. Second, *S. oneidensis* typically possesses an extraordinarily high content of cytochromes, which confers cells a characteristic pink or red color. The high content of cytochrome is suggestive of a high demand for iron, since iron is a co-factor of heme groups in the cytochromes [17]. Lastly, the majority of *S. oneidensis* genes are most similar to genes of *Vibrio cholerae*, the γ-proteobacterium causative of a diarrheal disease called cholera [18]. It has been established that iron acquisition genes of *V. cholerae* are required for successful colonization in animal models [19]. Since *Shewanella* species are fish pathogens and infrequent opportunistic human pathogens [20,21], it is possible that iron in *Shewanella* species plays a similar role to *V. cholerae* and function to signal the entry into the host.

Much of iron homeostasis in *S. oneidensis* remains to be elucidated. The Fur gene of *S. oneidensis* shares 74% identity with the *E. coli* homolog. Previous transcriptomic studies of the *fur* mutants under anaerobic and aerobic conditions indicate that it regulates a variety of biological processes including iron uptake [22,23]. A highly conserved Fur binding motif has also been predicted. These results are consistent with a role of *S. oneidensis* Fur in iron acquisition. However, responses of the *fur* mutant to iron depletion have not yet been examined. It is also unclear how disruption of the pleiotropic regulator Fur affects other cellular processes. In addition, although Fur is regarded as the master regulator for iron response, it has been shown in *Vibrio cholerae* and *Staphylococcus aureus* that a number of genes are regulated by iron independently of Fur [24,25]. It is interesting to investigate such systems in *S. oneidensis*.

In the present study, we performed physiological and transcriptomic studies to characterize the function of *S. oneidensis* Fur. Our results demonstrated that Fur played a role in iron acquisition and acid resistance response. We also discovered that a number of genes including genes related to anaerobic energy transport were regulated by iron rather than Fur. This work provides useful information in understanding the complicated complex molecular networks coordinating the bacterial response to iron.

## Results and discussion

### Phenotypic analyses of the *fur* mutant

**Figure 1:**
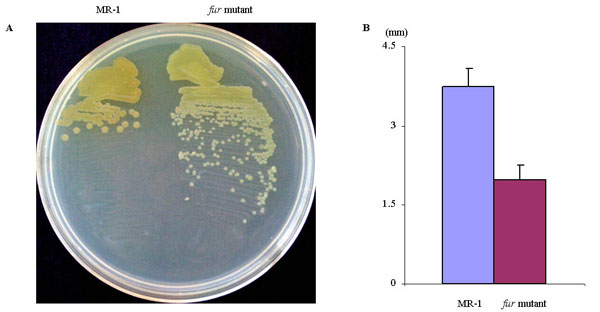
Growth of the *fur* mutant on solid LB medium. Bacteria were streaked on LB agar and then incubated at 30°C for two days. (B) Quantitative measurement of colony sizes. The average diameter of colonies, calculated from eight colonies of either strain, is shown.

A deletion mutant of Fur was constructed from wild-type *S. oneidensis* strain MR-1. The *fur* mutant formed smaller colonies than wild-type strain after two days' incubation at 30°C (Fig. 1), suggestive of a growth deficiency caused by Fur inactivation. Moreover, colonies of the *fur* mutant appeared paler in color than MR-1 colonies. Wild-type *S. oneidensis* colonies have characteristic reddish or pink pigments, which is attributed to high heme content in the cells [17]. The pale color of colonies of the *fur* mutant suggested that there was less heme, presumably due to the low level of intracellular iron as a heme co-factor. The low level of intracellular iron has been documented in an *E. coli fur* mutant [26].

**Figure 2:**
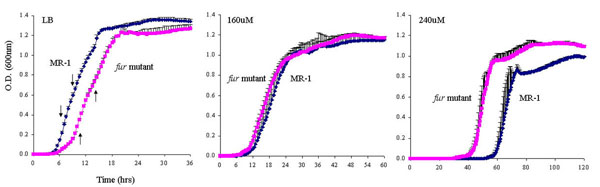
Comparative growth analyses of wild-type and the *fur* mutant. Growth curves for strains grown in liquid LB, LB-160uM and LB-240uM iron chelator are shown. Cells grown in mid-logarithmic phase were diluted 1/100 in fresh LB media and growth was monitored every thirty minutes during consecutive five days. Three replicates were used for both strains. The average growth and error bars are shown. The down arrows and up arrows indicate the range of mid-logarithmic phase of MR-1 and the *fur* mutant, respectively. The doubling time of MR-1 is 1.5 hrs. The doubling time of the *fur* mutant is 2.5 hrs.

To test the role of Fur in the response of *S. oneidensis* to iron, we grew the *fur* mutant and the wild-type strain in LB medium with different concentrations of the iron chelator 2, 2'-dipyridyl to create iron depletion conditions. LB medium contains ~17 uM iron, hence it is considered as an iron-rich medium [26]. In LB, the *fur* mutant displayed longer lag phase, slower growth rate at logarithmic phase and lower cell density at stationary phase than MR-1 (Fig. 2). When iron was depleted from the growth medium by addition of iron chelator, both strains clearly displayed growth inhibition. Interestingly, when both strains were grown with 160 uM iron chelator, no significant difference was detected between the mutant and MR-1. With 240 uM iron chelator, the *fur* mutant displayed a much shorter lag phase than MR-1 and reached a higher cell density at stationary phase, suggesting that the *fur* mutant better tolerated the iron depletion stress.

**Figure 3:**
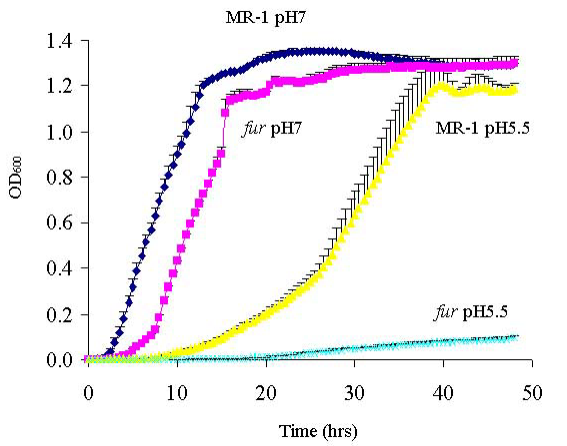
Growth of wild type MR-1 and the *fur* mutant strains at pH 5.5 and 7.0. Cells grown to mid-logarithmic phase were diluted 1/100 in fresh pH-buffered LB media. Growth was monitored every thirty minutes for two days. Three replicates were used for *S. oneidensis*. The average growth and error bars are shown.

Fur is a pleiotropic transcriptional factor controlling the expression of both iron-regulated and non-iron-regulated genes [9]. Fur has been implicated in acid tolerance response, since *E. coli* and *Salmonella typhimurium* harboring *fur* mutations are acid-sensitive [27,28]. Therefore, the acid tolerance of *S. oneidensisfur* mutant was examined. Wild-type and the *fur* mutant were grown in LB medium buffered at pH5.5 and 7 (Fig. 3). At pH of 5.5, the *fur* mutant but not wild-type strain had severe growth defect, demonstrating that the *fur* mutant was more sensitive to acidic condition.

### Microarray experiments

For microarray experiments, mid-log phase grown *fur* mutant was treated with iron chelator for one hour and then ferrous sulfate. The concentration of 160 uM iron chelator was used for iron depletion during the microarray experiments because growth was clearly inhibited, but not completely abolished at this concentration (Fig. 2). Samples were collected at multiple time points during iron depletion and repletion and used for global transcriptomic analyses. To allow for comparison of any pairs of samples, *S. oneidensis* genomic DNA was used as a common reference in each microarray experiment (see Methods and Materials for details). This strategy has been successfully employed in several studies [29-32]. In addition, since *S. oneidensisfur* mutant in previous studies was generated from a rifampin-resistant strain (DSP10) instead of wild-type *S. oneidensis* MR-1, we decided to repeat the microarray comparison between the *fur* mutant and wild-type under aerobic condition, using the *fur* mutant derived from MR-1. Therefore, MR-1 was grown in LB to mid-logarithmic phase, RNA was prepared and used for microarray experiments. This allowed for comparison of gene expression profiles of the *fur* mutant and MR-1 (see below). To evaluate the reliability of microarray data, quantitative RT-PCR was performed on a few selected genes (Additional file Additional file 1). A high Pearson correlation coefficient of 0.92 was observed between RT-PCR and microarray results.

### Fur-regulated genes

**Figure 4:**
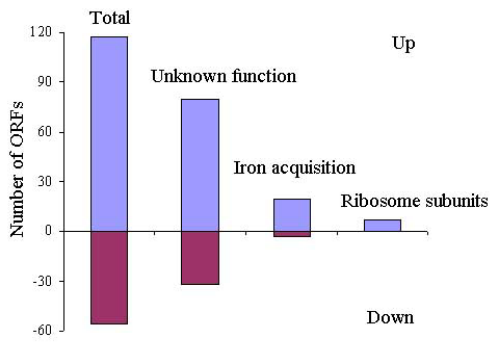
Up-expressed (up) and down-expressed (down) genes (|log_3_ ratio| ≥1 with *p*<0.05) in the *fur* mutant versus MR-1. The leftmost bar is the total number of up-expressed and down-expressed genes. Genes are grouped according to the function categories, as indicated by text.

Global gene expression profiles of the *fur* mutant and MR-1 grown in LB medium were compared. A total number of 118 and 56 genes were significantly up- and down- regulated, respectively (Fig. 4). As commonly seen in microarray datasets, a large portion of up- or down-regulated genes corresponded to genes with unknown function, indicating a much broader modulon than deduced by gene annotation alone. Among the genes with defined functions (Table 1), two groups of genes were most noticeable: genes encoding iron acquisition proteins and ribosome proteins. The high level induction of iron acquisition genes agreed with the expected role of Fur in regulating iron uptake and transport. It provided an explanation for the better growth of the *fur* mutant under iron-depleted condition (Fig. 2), since the up-regulation of iron acquisition genes imposed an advantage for rapid adaptation to the iron depletion. The induction of genes encoding ribosomal proteins was rather unexpected, as the *fur* mutant grown in LB medium displayed growth defects. It was possible that these genes were induced to accommodate the requirement to synthesize iron acquisition proteins induced by iron depletion. Alternatively, their induction could be due to cellular response to overcome the growth deficiency in LB. Notably, a conserved Fur binding motif could be identified in the promoter region of almost all of the iron acquisition genes in Table 1, while no ribosome subunit gene except SO0232 had a Fur binding motif in its promoter (data not shown). Therefore, it was likely that genes encoding ribosome proteins were not the direct targets of Fur. Lastly, several acid resistance systems have been characterized in γ-proteobacteria [33,34]. However, expression of components of these systems (e.g. Crp, RpoS, glutamate-, arginine- and lysine-decarboxylases) was not significantly altered in the *fur* mutant. Since the acid tolerance of the *fur* mutant was reduced, it was possible that these genes were regulated at post-transcriptional level, or the core components of these systems were not yet annotated in *S. oneidensis*, or *S. oneidensis* could employ a novel and yet unidentified mechanism for acid tolerance response.

**Table 1:** Representative genes that displayed significant up- or down-expression in the *fur* mutant

**Gene**	**Annotation**	**Ratio (*p* value)**
AlcA	siderophore biosynthesis proteinx	13.85 (1.4E-05)
CcmD	heme exporter protein CcmD	5.32 (0.01)
ExbB1	TonB system transport protein ExbB1	15.69 (4.6E-04)
ExbD1	TonB system transport protein ExbD1	12.56 (8.5E-04)
FeoA	ferrous iron transport protein A	5.96 (0.02)
HmuT	hemin ABC transporter, periplasmic hemin-binding protein	4.51 (2.4E-05)
HmuU	hemin ABC transporter, permease protein	5.46 (0.003)
HmuV	hemin ABC transporter, ATP-binding protein	5.46 (0.002)
HugA	heme transport protein	49.79 (4.7E-09)
IrgA	iron-regulated outer membrane virulence protein	8.90 (6.0E-04)
SO1482	TonB-dependent receptor, putative	5.39 (1.1E-06)
SO1580	TonB-dependent heme receptor	3.99 (4.1E-04)
SO3031	siderophore biosynthesis protein, putative	3.60 (1.0E-04)
SO3032	siderophore biosynthesis protein, putative	9.14 (1.4E-04)
SO3033	ferric alcaligin siderophore receptor	10.86 (1.2E-04)
SO3063	sodium: alanine symporter family protein	4.16 (1.6E-05)
SO3914	TonB-dependent receptor, putative	3.63 (0.002)
SO4743	TonB-dependent receptor, putative	3.82 (4.7E-04)
TonB1	TonB1 protein	21.92 (1.7E-05)
ViuA	ferric vibriobactin receptor	5.73 (2.9E-04)
Bfr1	bacterioferritin subunit 1	0.25 (0.04)
InfA	translation initiation factor IF-1	4.32 (0.001)
RplD	ribosomal protein L4	6.32 (4.5E-04)
RplK	ribosomal protein L11	28.83 (0.001)
RpmA	ribosomal protein L27	7.20 (0.005)
RpmF	ribosomal protein L32	3.63 (0.02)
RpmH	ribosomal protein L34	3.55 (0.02)
RpmI	ribosomal protein L35	7.62 (8.3E-04)
SO0401	alcohol dehydrogenase, zinc-containing	6.48 (4.0E-04)
HyaB	quinone-reactive Ni/Fe hydrogenase, large subunit	0.23 (0.04)
NqrD-2	NADH: ubiquinone oxidoreductase, Na translocating	0.27 (0.04)
SO1648	cold shock domain family protein	8.10 (0.02)
SO0130	putative protease	0.18 (0.05)
SO2426	DNA-binding response regulator	9.87 (4.2E-04)
SO0295	transcriptional regulator, LysR family	0.15 (0.03)
SO0577	sensory box histidine kinase/response regulator	0.18 (0.03)
SO2374	transcriptional regulator, LysR family	0.19 (0.03)
SO2498	sensory box protein	0.25 (0.04)
SO3059	formate hydrogenlyase transcriptional activator, putative	0.30 (0.05)
Rsd	regulator of sigma D	0.30 (0.05)

### Iron-responsive and Fur-independent biological processes

**Figure 5:**
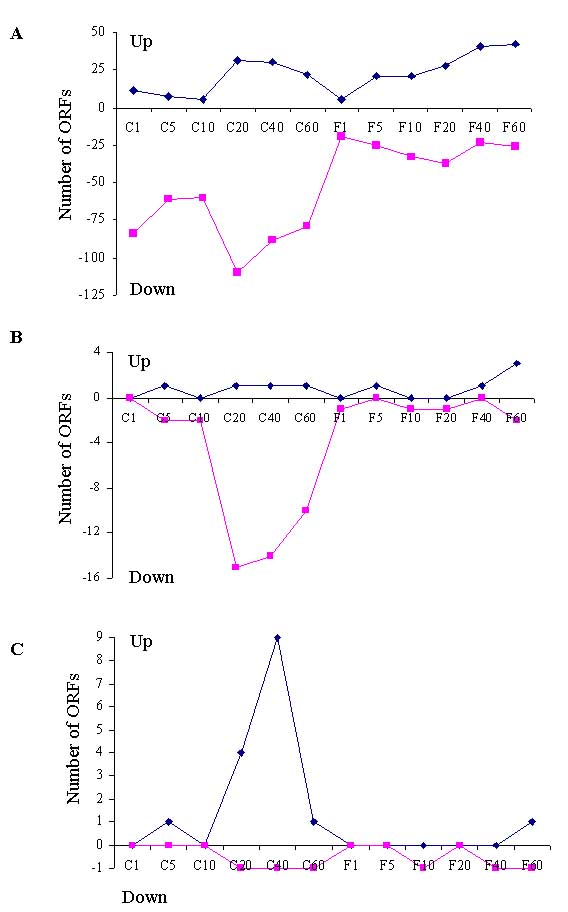
Numbers of genes predicted to be up-expressed or down-expressed (down) genes (|log_3_ ratio| ≥1 and p<0.05) of the *fur* mutant during the time course experiment. C1', C5', C10', C20', C40' and C60' are the time points of 1, 5, 10, 20, 40, and 60 minutes after adding iron chelator. F1', F5', F10', F20', F40' and F60' are the time points of 1, 5, 10, 20, 40, and 60 minutes after adding iron back to the iron-depleted culture. Total genes (A) and genes related to (B) anaerobic energy transport and (C) aerobic energy transport that are up-expressed or down-expressed are shown.

The microarray analyses of the *fur* mutant indicated that fewer genes was significantly regulated at early time points than later in both iron depletion and repletion, and there were more down regulated genes than up regulated genes during iron depletion (Fig. 5A). Few genes encoding iron acquisition or ribosome proteins were among those up or down regulated, suggesting that their regulation was strictly Fur-dependent. Meanwhile, a group of genes related to anaerobic energy transport was repressed by iron depletion (Fig. 5B), but not identified in Fur regulon shown above (Table 1). Therefore, this process was iron responsive but Fur independent. These genes include formate dehydrogenase and multiple *c*-type cytochromes that are involved in anaerobic energy respiration. For example, FccA is a fumarate reductase; CymA is a key protein controlling respiration with a variety of electron acceptors, while MtrC/OmcB is an outer membrane protein required for metal reduction. A logical explanation of the repression by iron is that under the tested condition, these genes are non-essential, iron-using proteins. Upon iron depletion, they are repressed to release previously sequestrated iron to increase free intracellular iron pool. This mechanism has been well documented in *E. coli*[4,35] as mediated by Fur and a small RNA RyhB. However, in *S. oneidensis* this biological process must be controlled by iron-responsive regulator(s) other than Fur. Another notable iron-responsive and Fur-independent process was aerobic energy transport, including TCA cycle enzymes malate dehydrogenase (Mdh), succinate dehydrogenase (SdhC), isocitrate dehydrogenase (SO1538) and citrate synthase (GltA). Other related genes include succinylarginine dihydrolase(AstB) and isovaleryl-CoA dehydrogenase (Ivd). Notably, in contrast to the genes involved in anaerobic energy transport, these genes were induced by iron depletion (Fig. 5C), suggesting that it was more energy-consuming for coping with iron starvation than the energetic need for normal growth.

### Gene co-expression network

Genes with the highest fold changes are not necessarily the most important genes for a given condition, since there usually is a dissociation between gene expression and physiological phenotype [36]. Moreover, a large number of significantly regulated genes are linked to hypothetical proteins. To gain more insights into their functions, a gene co-expression network was constructed (Additional file Additional file 2A). Microarray data have frequently been used for gene annotation based on the observation that genes with similar expression patterns are more likely to be functionally related, that is, “guilt by association”, despite the fact that co-expression does not necessarily indicate a causal relationship at the transcriptional level. This fact is exploited in the “majority-rule” method of network annotation in which a gene is annotated based on the most commonly occurring functions of its co-expressed partners.

**Figure 6:**
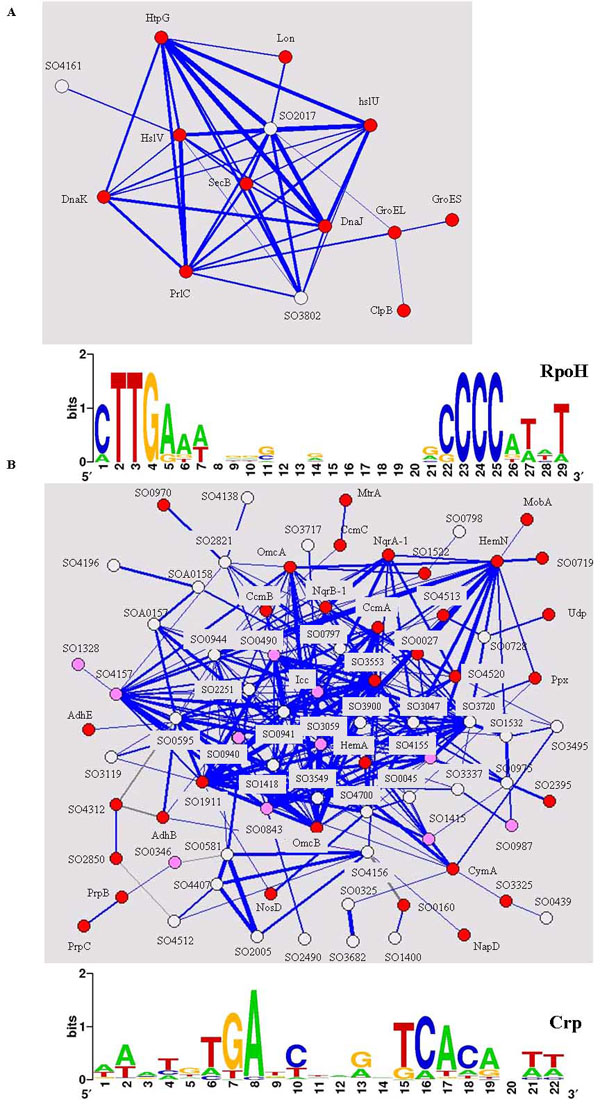
Two clusters of genes revealed in gene network. (A) Cluster of heat shock proteins. (B) Cluster of genes related to anaerobic energy transport. Each node represents a gene and the width of the line represents the correlation coefficient of two linked genes. Blue and gray lines indicate positive and negative correlation coefficients, respectively. Colors were assigned to nodes according to their functional categories: red represents known heat shock proteins in (A) or anaerobic energy transport in (B), white represents unknown genes and pink represents transcriptional regulators. The sequence logo of consensus sequence in the promoter regions of genes in the clusters was generated by the Weblogo program [45].

Functionally related genes were indeed grouped together in all clusters of the gene network that we constructed (Additional file Additional file 2A). For example, one cluster contains eleven genes encoding heat shock proteins (DnaK, DnaJ, HslV, HslU, HtpG, Lon, SecB, PrlC, ClpB, GroEL and GroES) and three unknown genes (Fig. 6A). These heat shock proteins function to accelerate turnovers of denatured or unfolded proteins and respond to multiple stress conditions [37]. Their identification in this study is expected since lack of iron as protein cofactor would impair the functionality of a number of proteins. Furthermore, it can be predicted based on the cluster that the unknown genes in this cluster might function in stress response. This prediction is further supported by their operon structures. SO4161 is immediately upstream of heat shock HslU and HslV and might form an operon together. Similarly, SO2017 could form another operon with the upstream gene encoding heat shock protein HtpG. Finally, when the Gibbs Motif Sampler [38] was employed to search the promoter regions of genes in this cluster for common sequence motif(s), a conserved RpoH (σ^32^) binding site was identified (Fig. 6A). In *E. coli*, RpoH directs the RNAP holoenzymes to specifically transcribed genes encoding heat shock proteins. Its presence in the promoter regions of SO2017 and SO4161 provides further evidence that these two genes play a role in stress response.

Another large cluster contains many *c*-type cytochromes (e.g. CymA, MtrA, MtrC and OmcA) and their related genes such as heme exporters and alcohol dehydrogenases (Fig. 6B). Many of them were considered as iron-responsive but Fur-independent, as discussed in the previous section. When the Gibbs Motif Sampler was applied, a palindromic sequence of “AAATGTGATCNNGNTCACA NTT” (Fig. 6B) was identified with statistical significance. This sequence is almost identical to the known binding motif of an *E. coli* global transcriptional factor Crp (AAA**TGTGA**TCTAGA**TCACA** TTT) [39]. In *S. oneidensis*, *crp* mutants are deficient in reducing Fe(III), Mn(IV), nitrate, fumarate and DMSO as electron acceptors [40]. The identification of a highly conserved Crp-binding site at the promoters of genes in this cluster provides an explanation for the phenotypes of *crp* mutants. Crp might act as a master regulator of anaerobic energy transport; its inactivation leads to repression of multiple branches of anaerobic energy transport pathway. Notably, the regulation of Crp to anaerobic energy transport is iron-responsive but Fur-independent, since most genes in this cluster shows little expression difference between the *fur* mutant and wild-type.

## Conclusion

The primary objective of the present study was to characterize the function of the pleiotropic regulator Fur and the iron response in *S. oneidensis*. Fur involvement in iron acquisition was evident, as shown by our physiological and transcriptomic studies. On the other hand, there were iron-responsive and Fur-independent systems (e.g. a large group of genes related to anaerobic energy transport). The identification of Crp binding site in Fig. 6B is consistent with the role of Crp in anaerobic energy transport and supports the notion that Crp is the master regulator of this bioprocess. The identification of a number of transcriptional factors in this cluster is worthy of future investigation. Over 200 genes of regulatory functions have been annotated in *S. oneidensis* genome (), yet only a small number of them have been examined experimentally. The involvement of the identified regulators in anaerobic energy transport, once verified, will contribute to the understanding of the complex branched respiratory systems in *S. oneidensis* and hence the potential utilization of *Shewanella* species to remediate U.S. DOE uranium-contaminated sites.

## Material and methods

### Bacterial strains and plasmids

To generate a *fur* deletion mutant from MR-1, a pDS3.1-*fur* suicide plasmid described in [23] was used. This plasmid contains the 5'- and 3'-flanking regions of Fur locus, with a 241-bp internal fragment of Fur locus removed. The plasmid was transformed into *E. coli* WM3064 strain prior to conjugal transferring into MR-1. Correct in-frame deletion was verified by sequencing PCR products using primers outside the DNA recombination region. The sequences of the primers are: (5'-GCA AGT ACA GCC GTT ATT CAC TCC-3') and (5'-GTA TCC AAA GGA TGC AAC TGG-3').

### Physiological studies

MR-1 and the *fur* mutant were grown to mid-log phase and diluted 1:100 into 300 ul fresh LB or M1 liquid medium. A Type FP-1100-C Bioscreen C machine (Thermo Labsystems) was used to measure the growth every thirty minutes. All physiological studies were done in triplicates so that average and standard error could be calculated. Different concentrations of iron chelator were prepared by dissolving 2, 2'-dipyripyl in water. For iron repletion, ferrous sulfate was used at the concentration of 200 uM.

### RNA preparation

Four biological replicates of MR-1 and the *fur* mutant were grown in LB to mid-log phase (OD_600_=0.6) and cells were collected. For the *fur* mutant, cells were also sampled at 1, 5, 10, 20, 40, and 60 minutes after adding 2, 2'-dipyridyl to a final concentration of 160 uM. Subsequently, ferrous sulfate was added to a final concentration of 200 uM. Cells were collected at 1, 5, 10, 20, 40, and 60 minutes thereafter. Total RNA was extracted using Trizol Reagent (Invitrogen) as described previously [23]. RNA samples were treated with RNase-free DNase I (Ambion, Inc.) to digest residual chromosomal DNA and then purified with RNeasy Kit (Qiagen) prior to spectrophotometric quantification at 260 and 280 nm.

### Microarray hybridization, scanning and quantification

cDNA was produced in a first-strand reverse transcription (RT) reaction and labeled with Cy5 dUTP (Amersham Biosciences) by direct labeling in the presence of random hexamer primers (Invitrogen). *S. oneidensis* MR-1 genomic DNA (gDNA) was amplified by Klenow (Invitrogen) and Cy3 dUTP was incorporated into the product (Amersham Biosciences). Fluorescein labeled probes were purified using a PCR purification kit (Qiagen). Slides were pre-hybridized at 50°C for about one hour to remove unbound DNA probes in a solution containing 50% (V/V) formamide, 9% H_2_0, 3.33% SSC (Ambion, Inc.), 0.33% sodium dodecyl sulfate (Ambion, Inc.), and 0.8 μg/μL bovine serum albuminin (BSA, New England Biolabs). Slides were hybridized at 50°C overnight with Cy5- and Cy3- labeled probes in the above solution, with 0.8 μg/μL herring sperm DNA (Invitrogen) replacing BSA to prevent random binding. Pre-hybridization and hybridization were carried out in hybridization chambers (Corning). Slides were then washed on a shaker at room temperature in the following order: 7 min. in 1x SSC, 0.2% SDS; 7 min. in 0.1x SSC, 0.2% SDS; and 40 sec. in 0.1x SSC.

A ScanArray Express Microarray Scanner (PerkinElmer) was used to scan slides. Fluorescence and background intensity were quantified using ImaGene 6 software (BioDiscovery, Inc.). All spots in which signal *vs*. background ratios were less than 3 were discarded.

### Microarray data analysis

GeneSpring 7.2 (SiliconGenetics) was used to remove outliers, perform LOWESS normalization and for analysis of statistical significance via a two-way t test for two independent conditions. To calculate the ratios over different time points, samples during iron depletion (namely, C1', C5', C10', C20', C40' and C60') were compared to samples without addition of chelator (C0'), and samples during the iron repletion (F1', F5', F10', F20', F40' and F60') were compared to C60' (1' is 1 min., 5' is 5 min., *etc*.). Genes with |log_3_ ratio| ≥1 with *p*<0.05 are considered significant. To construct a gene co-expression network from temporal gene expression profiles, a Random Matrix Theory (RMT) based algorithm as described in [41,42] was employed. Applying the RMT method to the microarray data revealed a Pearson correlation coefficient of 0.87 as the minimal threshold to construct gene co-expression network. Since gene co-expression networks are known to be hierarchical [43], higher thresholds have been used to recognize larger functional modules. As a result, nineteen modules were distinguished (Additional file Additional file 2A). The software Pajek [44] was used to visualize the gene co-expression network. To identify the common motif, the Gibbs Motif Sampler [38] was employed according to its manual. The promoter regions of genes in the heat shock cluster were scanned to identify RpoH-binding site. For Crp-binding site, the recursive model of the Gibbs Motif Sampler was used to scan the upstream intergenic regions.

### Real-time RT-PCR analysis

Real-time quantitative reverse transcription-PCR (RT-PCR) was performed as described previously [23], except that iQ SYBR green supermix (Bio-Rad) was used instead of SYBR green I.

### Microarray data accession

The microarray data are available at .

## Competing interests

The authors declare that they have no competing interests.

## Authors' contributions

YY contributed to the experimental design, conduction and analysis of the transcriptomic and physiological experiments and manuscript writing. DPH and ABP performed most of the transcriptomic experiments. FL contributed to the RMT analyses and data interpretation of the transcriptomic results. AVP is involved in revising the article critically. LW contributed to the microarray designing and fabrication. JZ contributed to the experimental design, data interpretation and manuscript revision. All authors read and approved the final manuscript.

## Supplementary Material

Additional file 1 (A) Sequences of quantitative RT-PCR (qPCR) primers used in this study; (B) Comparison of expression measurements by microarray and qPCR assays. Values > 1 and values < 1 indicate up- and down-regulation, respectively. Pearson correlation coefficient of 0.92 was obtained by comparing microarray data with qRCR data. Click here for file

Additional file 2 (A) The gene co-expression network derived from the microarray data of the *fur* mutant. Each node represents a gene and the width of line represents the correlation coefficient of two linked genes. Blue and gray lines indicate positive and negative correlation coefficients, respectively. Colors were assigned to nodes according to their functional categories per conventions used by TIGR (): red represents the major functional category of each module, as indicated by text; pink represents transcriptional regulator; white represents unknown genes and black nodes are genes whose association to other genes are not understood. The italic bold numbers are the cutoffs used to isolate modules. (B) Functional predictions from the gene co-expression network in (A). Click here for file
