# Supplementary figures and images for: Characterization of the Shewanella oneidensis Fur gene: roles in iron and acid tolerance response
Source: BMC Genomics. 2008 Mar 20;9(Suppl 1):S11. doi: 10.1186/1471-2164-9-S1-S11 (PMC2386053; doi:10.1186/1471-2164-9-S1-S11)

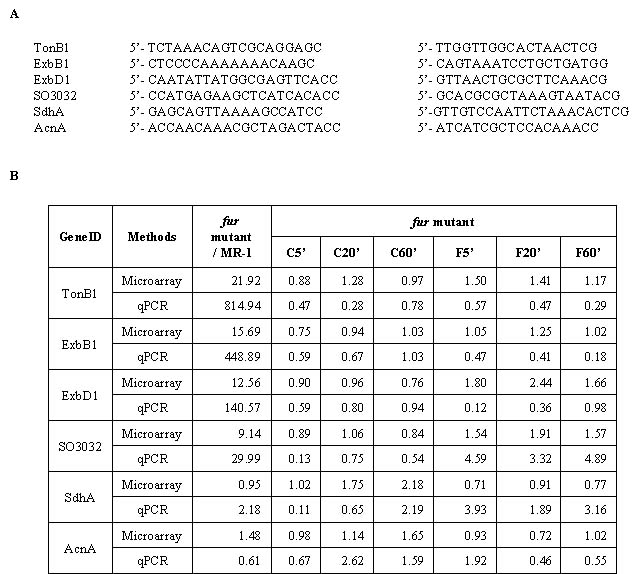

Supplement: Additional file 1 — (A) Sequences of quantitative RT-PCR (qPCR) primers used in this study; (B) Comparison of expression measurements by microarray and qPCR assays. Values > 1 and values < 1 indicate up- and down-regulation, respectively. Pearson correlation coefficient of 0.92 was obtained by comparing microarray data with qRCR data. [file 1471-2164-9-S1-S11-S1.JPG]

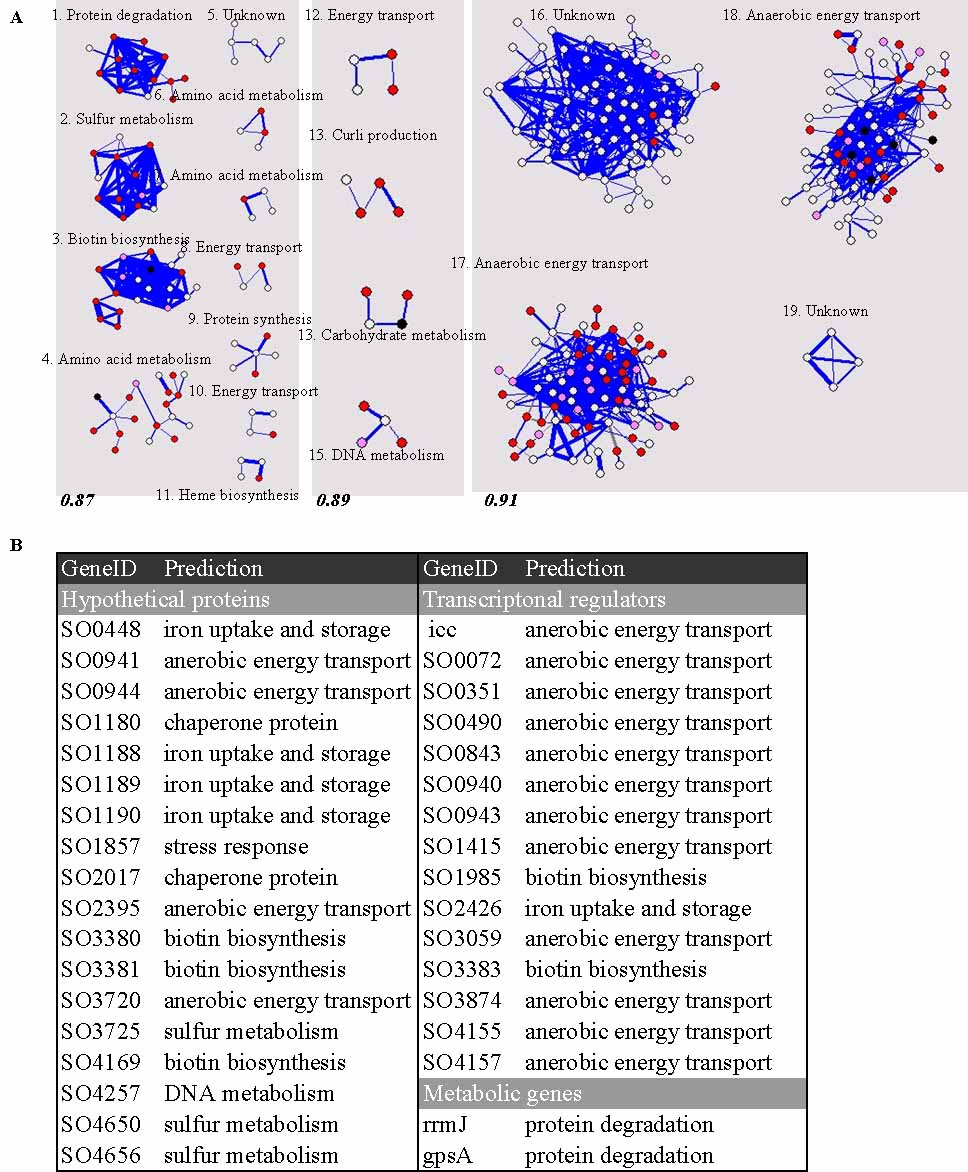

Supplement: Additional file 2 — (A) The gene co-expression network derived from the microarray data of the fur mutant. Each node represents a gene and the width of line represents the correlation coefficient of two linked genes. Blue and gray lines indicate positive and negative correlation coefficients, respectively. Colors were assigned to nodes according to their functional categories per conventions used by TIGR (): red represents the major functional category of each module, as indicated by text; pink represents transcriptional regulator; white represents unknown genes and black nodes are genes whose association to other genes are not understood. The italic bold numbers are the cutoffs used to isolate modules. (B) Functional predictions from the gene co-expression network in (A). [file 1471-2164-9-S1-S11-S2.JPG]
